# Photovoltaic and photocatalytic properties of bismuth oxyiodide–graphene nanocomposites†

**DOI:** 10.1039/c8ra07360k

**Published:** 2018-12-19

**Authors:** Levannie A. Mabuti, Ian Kenneth S. Manding, Candy C. Mercado

**Affiliations:** Department of Mining, Metallurgical, and Materials Engineering, University of the Philippines Diliman 1100 Quezon City Philippines ccmercado1@up.edu.ph

## Abstract

In this study, we evaluate the photovoltaic and photocatalytic properties of chemical vapor deposited bismuth oxyiodide (BiOI) and bismuth oxyiodide–graphene (BiOI–GR) nanocomposite thin films. The BiOI thin film has an average thickness of 574 nm and a bandgap of around 2 eV. The BiOI and BiOI–GR thin films exhibited nanoflake morphology. It was found that addition of graphene increases absorbance by causing vertical growth of nanoflakes, imparting anti-reflectance and light trapping properties. The photocatalytic activities of the thin films were evaluated by examining methylene blue (MB) degradation under visible light irradiation. BiOI–GR degraded 56.42% of MB in two hours while BiOI degraded 44.16%. Afterwards, FTO|BiOI|graphite|Al and FTO|BiOI–GR|graphite|Al solar cell devices were fabricated with photocurrent density values of 2.0 μA cm^−2^ and 2.7 μA cm^−2^, respectively. The improved properties of BiOI–GR are attributed to the anti-reflecting and light trapping properties of vertical BiOI–GR nanoflakes and the enhanced carrier separation due to graphene as an electron acceptor.

## Abstract 2

BiOI nanoflakes grown by air atmosphere chemical vapor deposition showed increased photocatalytic activity when deposited on a graphene coated substrate.

## Graphical abstract

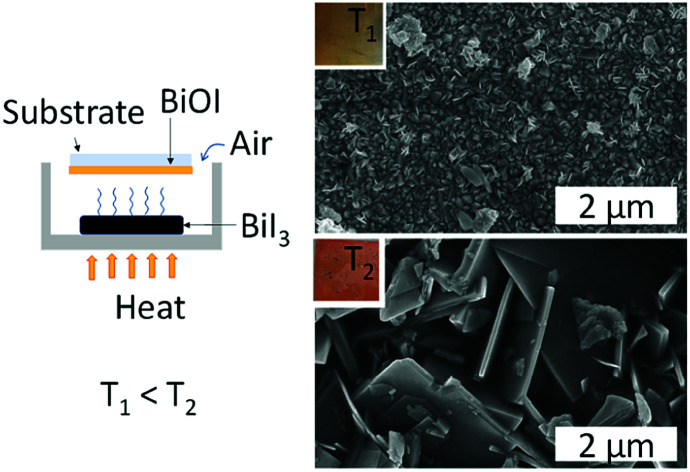

## 1. Introduction

Photovoltaics continues to be one of the technologies that contributes to energy and environmental sustainability.^1^ One of the developing PV technologies is the lead halide-based thin film solar cells; their high absorption coefficient, tunable band gap, long carrier diffusion lengths, and low fabrication costs make them a good candidate for next generation PVs. The efficiency of lead halide-based solar cells experienced a rapid rise from 3.8% to 22.1% over the course of seven years, illustrating their capability in the future to rival silicon solar cells in terms of efficiency.^2–5^

The advantage of lead-halide thin film solar cells was shown to be defect tolerance due to energetically shallow intrinsic defects of the Pb ns^2^ orbital.^6^ However, the development of lead halide-based “perovskite” solar cells (PbX PSCs) and their subsequent expansion for commercial use is hindered by the presence of the lead (Pb) in its two most promising compounds, methylammonium lead trihalides (CH_3_NH_3_PbX_3_) and formamidinium lead trihalides (H_2_NCHNH_2_PbX_3_),.^7–9^ Due to this, efforts were made in the exploration and development of other ns^2^-containing materials such as In^+^, Sb^3+^, and Bi^3+^. In 2015, Brandt *et al.* identified bismuth oxyiodide (BiOI), a non-toxic and low-cost material, as one of identified materials possessing the electronic structure necessary to replicate the defect-tolerance of PbX compounds.^10^ Through computations, Hoye *et al.* confirmed that BiOI is indeed tolerant to vacancies and antisite defects. Aside from being defect-tolerant, Hoye *et al.* also found that BiOI has a carrier recombination lifetime way longer than the 1 ns threshold for effective solar cell active materials.^11^ The only factor restraining its development into becoming the next-generation solar cell is its low efficiency, which peaked at 1.8%. Hoye *et al.,* however, claims that the theoretical limit for BiOI is 22% indicating its capability to rival Si solar cells and PSCs with further research and development.^11^

Photocatalysis converts solar energy into chemical energy used in various applications including hydrogen production through water splitting, hydrocarbon production from carbon dioxide, and degradation of environmental pollutants.^12^ Currently, titanium dioxide (TiO_2_) holds the distinction of being the most researched material for photocatalysis due to its excellent chemical stability, non-toxicity, economics, and high oxidizing activity. However, given these distinctive properties of TiO_2_, extensive technological use is limited a wide band gap of 3.2 eV, limiting its application in the visible light region.^13,14^ Practical applications of TiO_2_ are also constrained due to its low adsorption capacity to hydrophobic contaminants, high aggregation tendency, and difficulty of separation and recovery.^15,16^

Given these limitations, we studied novel nontitania-based photocatalysts for photocatalytic performance under visible light. Recently, bismuth oxyhalides (BiOX, X= F, Cl, Br and I) have drawn much interest for their potential application in photocatalysis.^17,18^ Among them, BiOI has the strongest absorption in the visible light and the narrowest band gap.^25^ Due to this and among other reasons, BiOI exhibits the highest photocatalytic activity compared to BiOBr and BiOCl in the degradation of organic pollutants such as methyl orange and crystal violet.^18–20^

The structure or composition of a material can be altered to obtain enhanced photovoltaic and photocatalytic properties. There are currently no previous studies on BiOI modification for photovoltaic applications. However, several studies have been done on the incorporation of other conductive materials to BiOI to increase its photoabsorbance and facilitate the separation of the photogenerated charge carriers for photocatalytic applications.^21^ One interesting carbonaceous material is graphene (GR): a monolayer of carbon atoms in a dense honeycomb structure, which has gained immense attention in the fields of research and industrial application due to its good conductivity, large surface-to-volume ratio, excellent chemical stability, high electron mobility, and excellent performance as electron acceptor and transport material.^22,23^ Several works have reported enhanced photocatalytic activity in water splitting, antibacterial application, and organic pollutant degradation by using photocatalysts combined with graphene.^24–26^ In the study of Liu *et al.*, novel BiOI–GR nanocomposites have been synthesized for the first time through a facile one-step hydrothermal method.^21^ On the other hand, Ye *et al.* synthesized BiOI nanostructure thin films *via* chemical vapor transport. Up to present, no study has reported on the preparation of BiOI–GR nanocomposite thin films *via* chemical vapor transport for solar cell and photocatalytic applications.^27^

This study investigates the potential of BiOI–GR thin film fabricated *via* chemical vapor deposition for photovoltaic and photocatalytic applications.

## 2. Results and discussion

### 2.1. Characterization

**Fig. 1:**
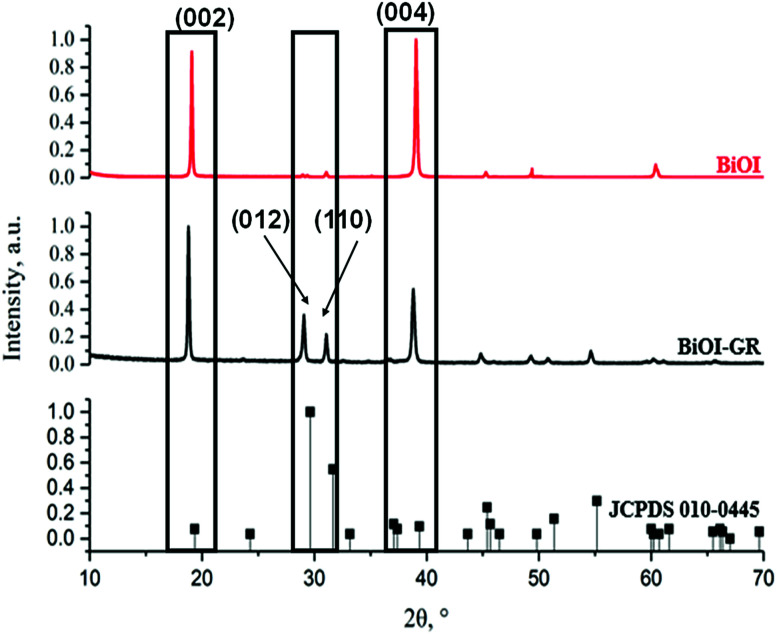
The XRD patterns of BiOI and BiOI–GR nanocomposite thin films deposited at 290 °C.

X-ray diffraction patterns of thin films of pure BiOI and BiOI–GR nanocomposite thin films deposited at 290 °C show peaks corresponding to tetragonal BiOI and no traces of BiI_3_, Bi_2_O_3_, Bi_5_O_7_I were detected (Fig. 1). In BiOI–GR films, characteristic peaks of graphene were not detected in the spectra due to relatively low amount and poor diffraction intensity. In Fig. 1, JCPDS 010-0445 is shown below the BiOI and BiOI–GR and a significant deviation between observed and reference peak intensities seen suggests the fabricated thin films exhibit an anisotropic texture. Pure BiOI featured pronounced (0 0 2) and (0 0 4) planes with both (0 1 2) and (1 1 0) planes having low intensities while diffraction from other peaks were not apparent. This indicates that most crystallites in BiOI are oriented with their (0 0 2) and (0 0 4) planes parallel to the substrate. This (0 0 1) textured crystalline structure is typically observed for BiOI thin films especially those deposited at relatively lower temperatures and oxygen partial pressure.^28^ For BiOI–GR, the planes (0 0 2) and (0 0 4) remain to be the dominant peaks with a significant increase in the intensity of other planes especially (0 1 2) and (1 1 0). This shows that most crystallites in BiOI–GR start to vary in orientation becoming more randomly oriented compared to pure BiOI film.

**Fig. 2:**
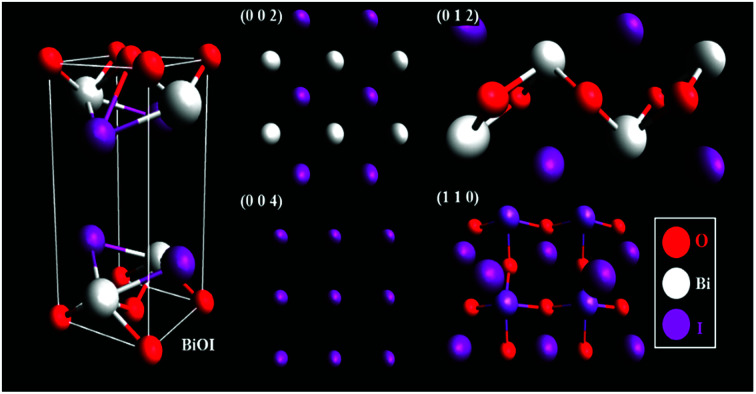
Crystalline structure and BiOI (left) and the dominant planes in BiOI–GR (right).

An examination of the planes in the BiOI lattice using representative slabs of the planes (0 0 2), (0 0 4), (0 1 2), and (1 1 0) shown in Fig. 2, for BiOI–GR, the oxygen-rich (1 1 0) and (0 1 2) planes grew parallel to the substrate plane due to the tendency of graphene to physically adsorb O_2_ molecules into its surface, effectively making it a nucleation site for the growth of the (1 1 0) and (0 1 2) planes.^29^ Conversely, the absence of graphene nucleation sites during the deposition of pure BiOI resulted into faint (1 1 0) and (0 1 2) peaks.

**Fig. 3:**
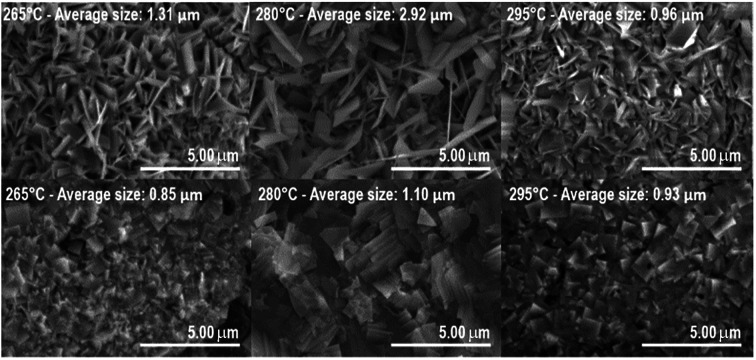
SEM images of BiOI–GR (top) and pure BiOI (bottom) thin films deposited at 265 °C, 280 °C, and 295 °C (L–R).

SEM images of samples deposited at 265 °C, 280 °C, and 295 °C are shown in Fig. 3. The samples exhibited a nanoflake morphology for both BiOI and BiOI–GR. On one hand, the nanoflakes of pure BiOI grew parallel to the surface. Since majority of BiOI crystallites are oriented with their (0 0 2) and (0 0 4) parallel to the substrate surface, the top and bottom surfaces of BiOI nanoflakes can be assigned to either of those planes. On the other hand, the nanoflakes of BiOI–GR grew perpendicular to the substrate surface, a phenomenon typically observed on thin films fabricated through vapor deposition on a textured substrate.^30,31^ During the initial stages of BiOI–GR nanoflake formation, nucleation occurred at sites with graphene, which ensued outward crystal growth. However, the substrate is not fully covered in graphene, thus not all the nanoflakes nucleated on graphene, some, instead, grew from the substrate. Despite not nucleating on graphene, these nanoflakes still grew vertically due to restriction of lateral growth along the substrate surface because of the graphene layer. After some time, crystallite growth along that direction was impeded. This results to the more diverse texture of the BiOI–GR film.

**Fig. 4:**
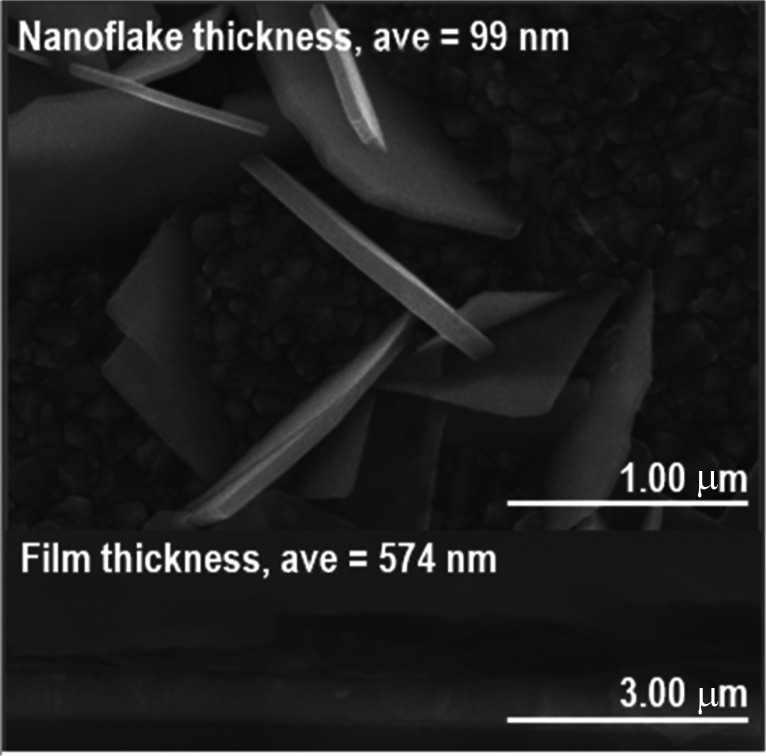
FESEM image the BiOI thin film nanoflakes on FTO (top) and their cross-section (bottom).

**Fig. 5:**
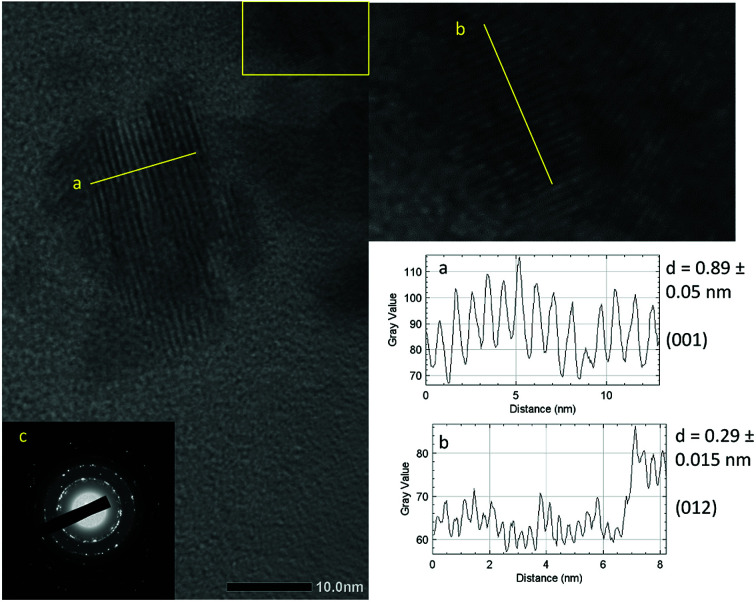
TEM images of the nanoflakes showing the lattice spacings for the (a) 001 (b) 102 planes. The corresponding line profiles were used to determine the distances. (c) SAED image showing high level of crystallinity.

As shown in Fig. 3, the crystallite size of thin films deposited in each temperature were determined. One can see that for both BiOI and BiOI–GR thin films, the thin films with the largest nanoflake were deposited at 280 °C. At this temperature, surface diffusion and growth of BiOI nuclei occurred which resulted to larger crystallites compared to crystallites of the thin film deposited at 265 °C. As the temperature was increased to 295 °C, densification of the thin film occurred.^32,33^Fig. 4 shows high magnification FESEM images of the surface and cross section of BiOI samples deposited on FTO at 290 °C. The average thickness of the deposited BiOI thin film was 574 nm whereas the thickness of one nanoflake is 99 nm. The ultrasonically dispersed nanoflakes were imaged in transmission electron microscope to show the order of the atoms to be long range. The major crystal plane is the 001 with interplanar spacing of around 0.9 nm and matches well with the lattice parameter *c* of BiOI. The other visible plane is given by spacing of 0.3 nm and corresponds to the 012 plane of BiOI. Electron diffraction showed the TEM sample to be intermediate between polycrystalline and single crystalline shown by the apparent rings and points in the selected area electron diffraction (SAED) image (Fig. 5).

**Fig. 6:**
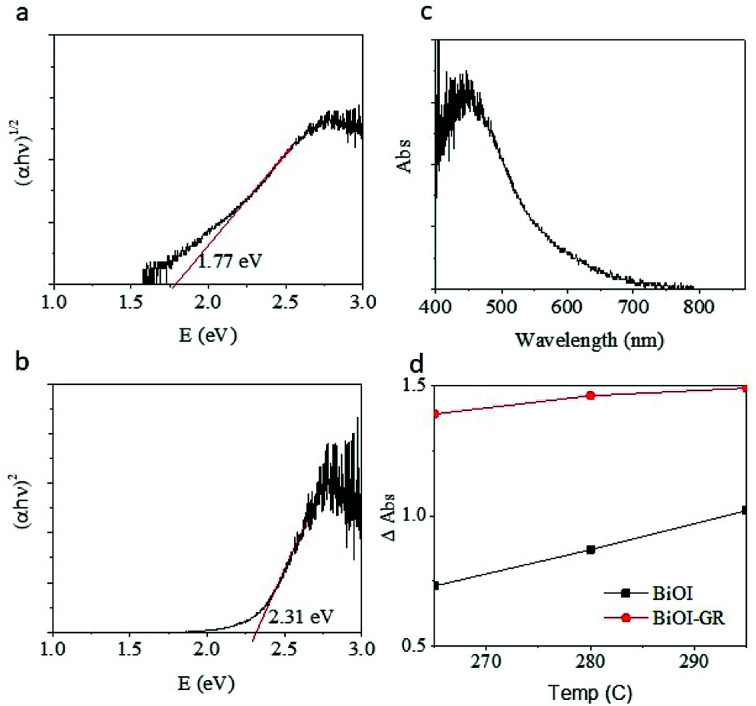
Tauc plot of BiOI thin film deposited at 290 °C (a) indirect (b) direct. (c) Absorbance of the BiOI film (d) relative absorption of BiOI and BiOI–GR.

The visible absorbance of the BiOI and BiOI–GR samples deposited at 265 °C (BiOI-265), 280 °C (BiOI-280), and 295 °C (BiOI-295) showed that absorbance increases by addition of graphene and increasing deposition temperature (Fig. 6d). The graphene-induced absorbance enhancement may be attributed to the morphology of the BiOI–GR nanoflakes. As seen in Fig. 3, BiOI–GR thin films exhibit vertical growth, imparting light trapping and anti-reflectance properties which prevent loss through transmission and reflection, respectively. If a thin film transmit light which the substrate inadvertently reflects, the vertically-grown nanoflakes of BiOI–GR traps the reflected light and absorbs it. This is not the case for the horizontally-grown BiOI nanoflakes. A similar event occurs when nanoflakes reflect incident light themselves. Much like in the previous scenario, BiOI–GR can prevent loss by trapping and absorbing reflected light whereas the BiOI nanoflakes cannot. Several studies have been done on the anti-reflective and light trapping properties of vertically-grown nanoflakes such as the study of Maurya *et al.* on zinc oxide nanoflakes and the study of Yin *et al.* on cadmium sulfate nanoflakes.^34,35^ However, the anti-reflectance and light trapping properties of BiOI are yet to be studied more extensively.

The increase in absorbance with deposition temperature is attributed to the effect of temperature on the texture of the thin film. For BiOI-295, large crystallites were formed at random orientation around the substrate perpendicular making a thin film with considerable roughness. For BiOI-280, even larger crystallites were formed but were stacked upon each other. Lastly, for BiOI-265 small crystallites formed, making the thin film relatively smooth compared to BiOI-295. Fig. 3 showed the progressive increase of roughness with the increase in temperature for BiOI. BiOI-295 has the highest roughness and is more effective in light trapping and preventing reflection of incident light. Moreover, BiOI-280 follows BiOI in terms of roughness and therefore also absorbance. BiOI-265 being the smoothest among the three exhibited the lowest absorbance. Significant roughness was observed in all BiOI–GR samples. For BiOI–GR-265, the nanoflakes were small and therefore can only trap light reflected at low angles with respect to the substrate. BiOI–GR-280 had significantly bigger nanoflakes and therefore had higher spacing between the nanoflake surfaces. The crystallites of BiOI–GR-280 can only trap light reflected at low angles relative to the substrate due to the large spacing between nanoflakes. BiOI–GR-295 had nanoflake size that is in between that of BiOI–GR-265 and BiOI–GR-280. This proved to be the optimal size because it was tall enough to trap high angle reflected light and yet small enough not to cause high spacing between nanoflake surfaces. Fig. S7† illustrates how this light trapping can occur in each of the BiOI–GR samples.

The addition of graphene can cause a redshift in the absorbance spectra when, according to a study by Liu *et al.*, a decrease in band gap energy is seen on BiOI–GR nanocomposites due to Bi–O–C bond formed by the bonding of unpaired π electrons on graphene with the Bi atoms of BiOI.^21^ The individual absorbance spectra of the samples with and without graphene showed a redshift in the onset of the peak, however, due to UV absorbance of the substrate and scattering by the sample the peak of absorbance was not seen (Fig. S9†). The light absorption in the lower energies of BiOI–GR is caused by the optical absorption of graphene.

The potential of BiOI and BiOI–GR as photocatalyst and photovoltaic material was determined from the calculation of the bandgap. The bandgap of BiOI-295 was determined using the Tauc plot as shown in Fig. 6(a–c). Extrapolating from the distinctly linear region of the plot, it was found that the bandgap of the deposited BiOI was 2.31 eV (using direct) and 1.77 eV (for indirect) transition coefficients and agrees with reported values, the yellow-orange color of the film, and the absorbance peak being at 584 nm.^36^ Most importantly, a bandgap of around 2 eV is at par with other semiconductors being used in the photovoltaic and photocatalytic devices currently.^11^

**Fig. 7:**
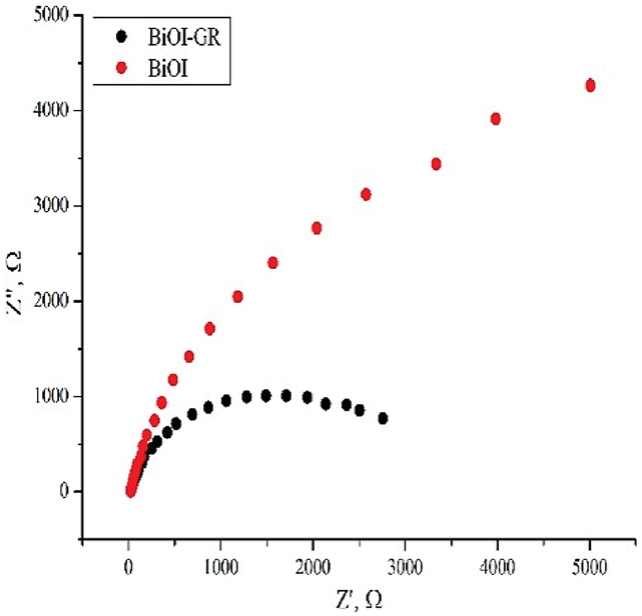
Impedance spectra of BiOI and BiOI–GR 320 °C thin films.

Fig. 7 shows the Nyquist plot which displays the measured impedance of BiOI and BiOI–GR thin films for forward bias of 0.15 V under illuminated condition. These films were deposited at 320 °C to ensure uniformity of surface coverage. From here, the *R*_ct_ of BiOI–GR is smaller than BiOI when modelled as a parallel resistor and capacitor. BiOI–GR indicates higher tendency of the photogenerated charge carriers to be transferred to the GR coated substrate. In photocatalysis and photovoltaic applications, lower *R*_ct_ contributes to electron transfer and enhanced fill factor and PCE.^37,38^

### 2.2. Photovoltaic properties

**Fig. 8:**
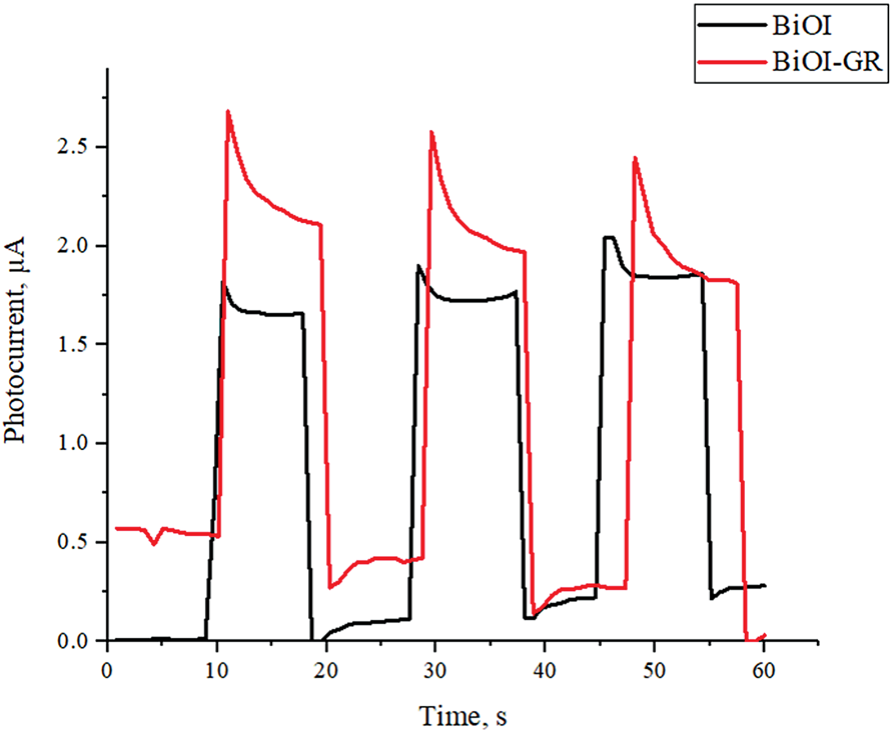
Photoresponse of the solar cell devices.

The assembled FTO|BiOI|graphite|Al and the FTO|BiOI–GR|graphite|Al solar cell devices were subjected to photocurrent measurement by irradiating the devices with visible light for one minute. Fig. 8 shows the measured photocurrent generated by the devices under visible light irradiation. The photocurrent of the two devices were compared and it was found that the solar cell with BiOI–GR absorber has slightly higher photocurrent generated as compared to the device with BiOI absorber. The solar cell device with BiOI–GR absorber produced photocurrent of 2.7 μA cm^−2^ while the device with BiOI absorber generated 2.0 μA cm^−2^. The higher photocurrent generated by device with BiOI–GR absorber and is attributed to the charge carrier separation mentioned earlier. Photocurrent generation is limited by electron–hole recombination and photocurrent response is related to the lifetime of complete charge separated state.^39,40^ The graphene in BiOI–GR thin film served as electron acceptor, hence photogenerated charge carrier recombination is prevented and the carrier life time of the charges were extended, hence generating higher photocurrent. This is illustrated in Fig. S8.† The enhancement in photocurrent density is also attributed to the enhanced absorbance with the addition of graphene, both of which enhances free electron generation.

The photocurrent density values obtained for both BiOI and BiOI–GR are far below the theoretical limit for BiOI which is 11 mA cm^−2^.^41^ The low-power lamp used in PV testing lead to low free electron generation and thus low photocurrent density. The standard power for light source in photocurrent density testing is 100 W cm^−2^ whereas the light source used was estimated to have a power rating of 10 W cm^−2^. However, our objective was to compare the performance with the addition of graphene and we have kept the testing and other parameters constant.

### 2.3. Photocatalytic properties

**Fig. 9:**
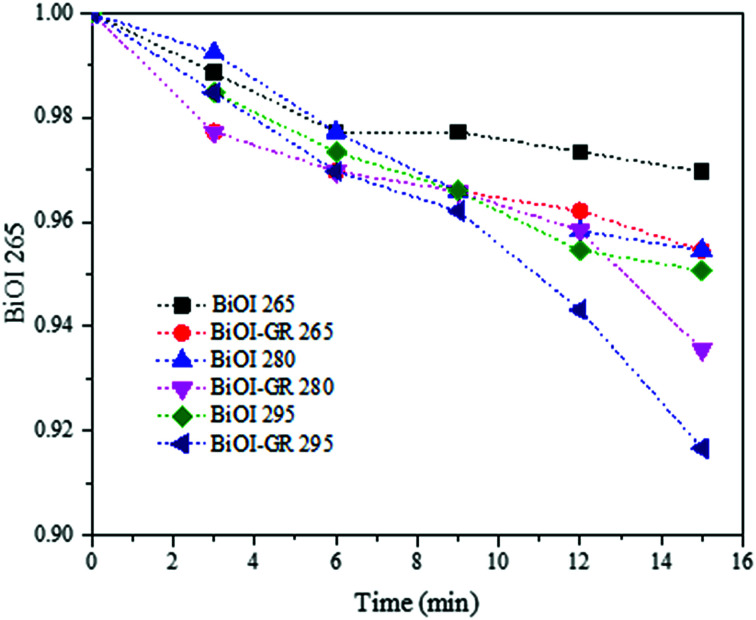
Photocatalytic activities of BiOI and BiOI–GR thin film samples deposited at 265 °C, 280 °C, and 295 °C.

The photocatalytic activities of BiOI and BiOI–GR thin films were evaluated by measuring the degradation of MB in an aqueous solution under visible light and are illustrated in Fig. 9. Thin films deposited at temperatures 265 °C, 280 °C, and 295 °C were initially subjected to photocatalytic activity test and BiOI-295 exhibited the highest photocatalytic activity among all pure BiOI samples whereas BiOI–GR-295 exhibited the highest photocatalytic activity among all BiOI–GR samples. This can be attributed to the high absorbance of BiOI-295 and BiOI–GR-295 compared to other films processed at different temperatures. The increase in photogenerated carriers also improved the photocatalytic system as shown by increase in MB degradation rate.

**Fig. 10:**
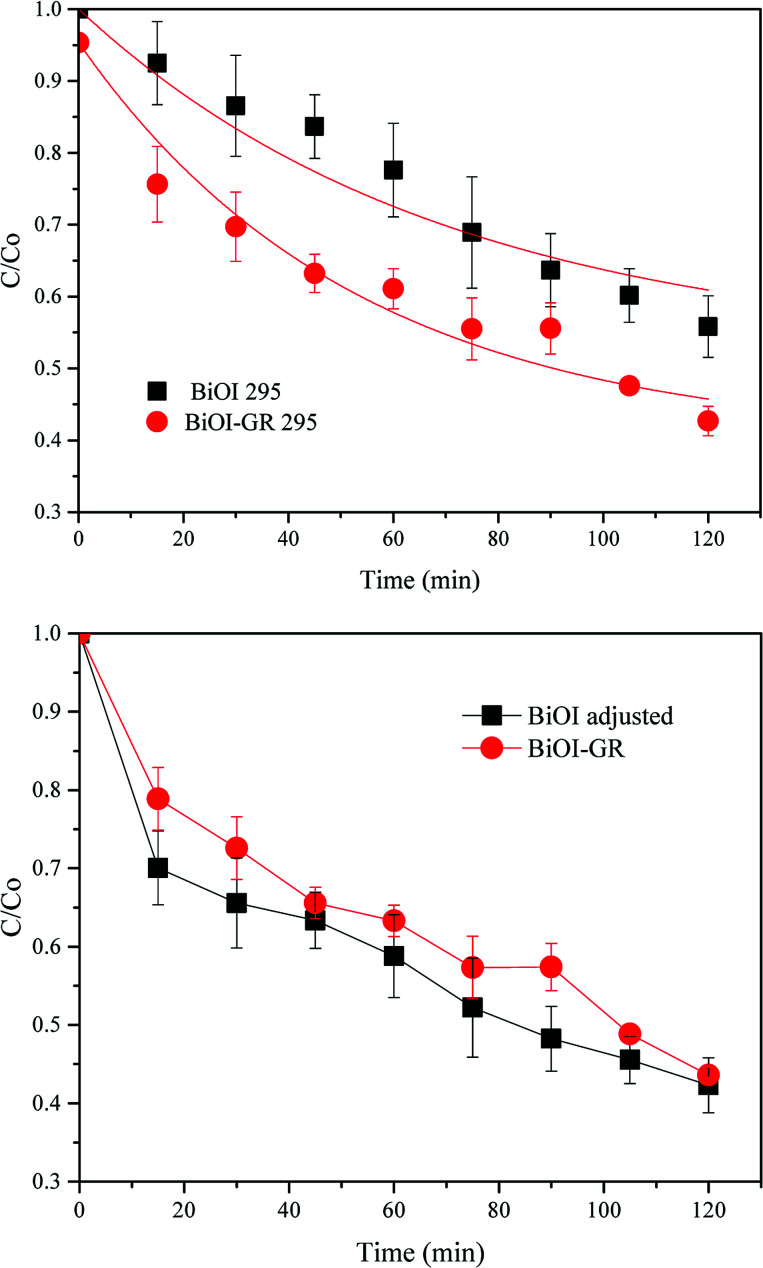
Long term photodegradation of MB with (295 °C deposited) BiOI and BiOI–GR thin film samples before (above) and after (below) correction for the surface area difference due to the roughness and nanoparticle morphology.

Due to the differences in structures, it is still interesting to determine the effect of both BiOI-295 and BiOI–GR-295. These were therefore used as photocatalysts for the degradation of MB under visible light for two hours. As shown in Fig. 10, BiOI–GR removed 12.26% more MB compared to pure BiOI over the course of 2 hours, pure BiOI degraded 44.16% MB while BiOI–GR degraded 56.42%. This shows improvement of BiOI–GR as a photocatalyst compared to pure BiOI which can be attributed to their morphology as well as charge separation.

From the characterization of BiOI in the earlier section, the roughness of BiOI and BiOI–GR vary greatly with BiOI having 001 planes aligned horizontally and for BiOI–GR, vertically. Taking this into consideration, the measured decrease in intensity was normalized for roughness and the results showed that BiOI will have slightly higher photocatalytic effect that BiOI–GR (Fig. 10) when absorbance and surface area are made equal assuming charge transport is comparable in both structures.

Photocatalysis involves three main processes: absorption of incident photons, charge separation and transfer, and surface charge utilization. The photogeneration and separation of electron–hole pairs are the primary factors affecting a photocatalytic reaction.^42^ When two materials are combined, electrons would flow from the material with higher Fermi energy to the material with lower Fermi energy to align the Fermi energy levels at their interface.^43,44^ In terms of work function, graphene has higher work function compared to BiOI, hence photogenerated electrons would flow from BiOI to graphene to adjust their Fermi energy levels. This results into the formation of a Schottky barrier at the BiOI–graphene interface.^45^ The Schottky barrier can capture electrons from BiOI to graphene which spatially separates electrons from BiOI and effectively enhance separation of the photogenerated electron–hole pairs.^20^ Thus, for BiOI–GR, graphene served as acceptor of the photogenerated electrons from BiOI. Recent papers have given the energy levels of the BiOI conduction band and graphene work function to be as shown in Fig. S10.† The charge transfer is slightly uphill in energy and could be the reason why the increase in photocurrent is not as large with the addition of graphene layer. Although this is the case, an improvement is seen in photocatalytic property due to good mobility of electron in graphene leading to several spatially separated locations for electrons. These electrons (in graphene) and holes (in BiOI) lead to more sites for reactions that both contribute to the degradation.

The absorbance spectra of methylene blue during degradation is shown in Fig. S6.† The intensity of the red absorbance peaks, one at 614 nm and another at 668 nm, decreases simultaneously as the exposure time is increased. In other studies of methylene blue degradation, the relative peak intensities vary as the demethylation progress, first a decrease of the 614 nm band relative to the 668 nm, then decrease of the 668 nm and 614 nm, then shifting of the peak from 668 nm to 620 nm.^46^ This spectral change corresponds to the *N*-demethylation of MB in a step wise manner due to ·OH or h_v_^+^ attack.^46^ Another study on decomposition of MB in the presence of In_2_S_3_ showed decolorization progress similar to this study which was proposed to be due to oxidative decomposition by electron injection from MB to In_2_S_3_.^47^ In the paper by Liu, *et al.*,^21^ The enhanced photocatalytic activity of BiOI–GR is attributed to the addition of graphene which prevented the photogenerated carrier recombination. The electrons could be transferred to surface-absorbed oxygen and form activated ·O_2_^−^ which further react with H^+^, producing hydroxyl radical ·OH. Accumulated holes in the valence band of BiOI could also react with H_2_O to produce hydroxyl radical ·OH, which is a photooxidation process. Both processes generate ·OH which can degrade MB.^21^ In a comprehensive study by Lee, *et al.*, the generated main active species are ·O_2_^−^ with the occurrence of ·OH at a lower probability in the photocatalytic degradation of crystal violet.^48^

The photocatalytic activity of BiOI–GR in this study, however, is lower than previous studies made on BiOI–GR photocatalysts. Liu *et al.* reported that BiOI–GR nanocomposite photocatalysts synthesized *via* hydrothermal method removed ∼62% of methyl orange in 2 hours due to the difference in illumination mentioned earlier.^21^ Differences in degradation mechanism can account for the differences in photocatalytic degradation performance and necessitates further studies on the photocatalytic degradation in BiOI/MB and other organic dye system.

## 3. Experimental

### 3.1. Materials

Bismuth iodide (BiI_3_) with 98% purity was obtained from Shanghai Macklin Biochemical Co. Ltd. Ultra-high-quality conductive graphene flake powder was obtained from AZ Laboratories. Fluorine-doped tin oxide (FTO) coated glass slide with dimensions 100 mm × 100 mm × 3 mm and a surface resistivity of 10 Ω sq^−1^ was obtained from Sigma-Aldrich Co. All reagents were used as received and not subjected to purification methods.

### 3.2. Thin film fabrication

All glass slides and FTO conductive glass used, measuring 2 cm × 5 cm, were subjected to ultrasonication once in soap solution for 15 minutes, and then twice in distilled water for 5 minutes. They were subsequently washed with ethanol and isopropanol. Graphene was deposited on the glass slide and FTO by spin coating 100 μL of the prepared graphene on ethanol solution for 20 s at 3500 rpm. The solution was prepared by adding 0.30 g of graphene on 10.0 mL ethanol and sonicating it for 30 minutes. BiOI–GR thin films were fabricated *via* chemical vapor deposition route by preparing 0.3 g of BiI_3_ into the ceramic crucible. With the graphene-coated glass slide or FTO on top of it, the crucible was heated on a hot plate in their designated temperatures for 3 hours. FTO samples were heated at 290 °C, 305 °C, and 320 °C whereas glass slide samples were heated at 265 °C, 280 °C, and 295 °C. This process was repeated for BiOI thin films without the spin coated graphene.

### 3.3. Characterization of BiOI and BiOI–GR thin films

The crystalline structure of the BiOI and BiOI–GR thin films deposited at 290 °C was characterized by using Rigaku Automated Multipurpose X-ray Diffractometer over the scanning range 2*θ* = 10–80°. The morphology of the BiOI and BiOI–GR thin films deposited at 265 °C, 280 °C, and 295 °C was characterized using SE3000 Scanning Electron Microscope using a 15.0 kV acceleration voltage. The film thickness of the BiOI sample deposited at 320 °C was determined using Hitachi 8230 Cold FESEM. TEM with SAED was used to determine the surface planes and crystallinity. ImageJ was used to analyse the images.^49^ To obtain their absorbance, the BiOI and BiOI–GR samples deposited at 265 °C, 280 °C, and 295 °C was subjected to UV-Vis spectroscopy using an Ocean Optics Modular Spectrometer Model Flame-S-UV-Vis-ES.

The charge transfer resistance of the BiOI and BiOI–GR samples deposited at 320 °C was characterized through electrochemical impedance spectroscopy (EIS) on a Metrohm Autolab PGSTAT 128N through the three-electrode set-up. The set-up used 1.0 M Na_2_SO_4_ as the electrolyte. Moreover, platinum was used as counter-electrode, Ag/AgCl was used as reference electrode, and the samples were used as working electrode. During testing, 1 cm was maintained between the sample and the counter electrode. EIS testing was done under illumination. A 10 W G.E. LED Coast Floodlight LFLD10WDL was used for this purpose.

### 3.4. Photocatalytic activity testing

A preliminary photocatalytic activity test was conducted to determine which among the three deposition temperatures produces the thin film which exhibits the highest photocatalytic activity. The photocatalytic activity of the BiOI and BiOI–GR thin films deposited at 265 °C, 280 °C, and 295 °C were evaluated by monitoring the progressive decrease in the amount of dye in the methylene blue solution through photocatalytic treatment under visible irradiation. The BiOI and BiOI–GR thin films were placed in 1 × 10^−5^ M MB and illuminated with 10 W G.E. LED Coast Floodlight LFLD10WDL for 15 minutes. Starting at time = 0, the absorbance of the MB solution was taken every 3 minutes. During preliminary testing, BiOI and BiOI–GR thin films deposited at 295 °C degraded the most amount of MB in 15 minutes. An extended photocatalytic activity test was then conducted to these samples with 3 replicates each. The same equipment and set-up were used; however, the testing was prolonged to 2 hours and absorbances were taken every 15 minutes starting at time = 0.

### 3.5. Device fabrication and testing

The photovoltaic properties of the BiOI and BiOI–GR solar cells were evaluated by measuring the photocurrent generated by the devices. To measure the photocurrent, the devices were irradiated with visible light for 1 minute using the 10 W G.E. LED Coast Floodlight LFLD10WDL as light source. At approximately ten-second intervals, the light source was turned on and then off. The photocurrent was recording using a Fluke 8808A Digital Multimeter.

## 4. Conclusions

BiOI and BiOI–GR nanoflake thin films were successfully fabricated *via* chemical vapor deposition for photovoltaic and photocatalytic applications. BiOI nanoflakes grew horizontally whereas BiOI–GR nanoflakes grew vertically. The difference in morphology between BiOI and BiOI–GR heavily influences the absorbance of the thin film because the vertically grown nanoflakes impart light trapping and anti-reflectance properties to the thin film, enhancing their absorbance. Moreover, thin films deposited at 295 °C exhibited superior absorbance as result of the optimal nanoflake size formed at this temperature. In FTO|BiOI–GR|graphite|Al photovoltaic cells, the photocurrent density of BiOI–GR is slightly higher than pure BiOI counterparts, FTO|BiOI|graphite|Al. BiOI and BiOI–GR performance in photocatalytic degradation of MB was higher with respect to percent MB degraded however, considering the total surface area of each film resulted in a comparable performance for both films with the pure BiOI showing higher MB degradation.

## Conflicts of interest

There are no conflicts to declare.

## Supplementary Material

RA-008-C8RA07360K-s001

## References

[cit1] Schiermeier Q., Tollefson J., Scully T., Witze A., Morton O. (2008). Nature.

[cit2] Kojima A., Teshima K., Shirai Y., Miyasaka T. (2009). J. Am. Chem. Soc..

[cit3] Hsieh T., Wei T., Wu K., Ikegami M., Miyasaka T. (2015). Chem. Commun..

[cit4] Ryu S., Noh J., Jeon N., Chan Kim Y., Yang W., Seo J., Seok S. (2014). Energy Environ. Sci..

[cit5] Yin P., Ling T., Lu Y., Xu Z., Qiao S., Du X. (2015). Adv. Mater..

[cit6] Stranks S., Eperon G., Grancini G., Menelaou C., Alcocer M., Leijtens T., Herz L., Petrozza A., Snaith H. (2013). Science.

[cit7] Abate A. (2017). Joule.

[cit8] Docampo P., Hanusch F., Giesbrecht N., Angloher P., Ivanova A., Bein T. (2014). APL Mater..

[cit9] Wang M. (2017). Sci. Bull..

[cit10] Brandt R., Stevanović V., Ginley D., Buonassisi T. (2015). MRS Commun..

[cit11] Hoye R., Lee L., Kurchin R., Huq T., Zhang K., Sponseller M., Nienhaus L., Brandt R., Jean J., Polizzotti J., Kursumović A., Bawendi M., Bulović V., Stevanović V., Buonassisi T., MacManus-Driscoll J. (2017). Adv. Mater..

[cit12] Ameta R., Ameta S. (2016). Photocatalysis.

[cit13] Ishikawa A., Takata T., Matsumura T., Kondo J., Hara M., Kobayashi H., Domen K. (2004). J. Phys. Chem. B.

[cit14] Zaleska A. (2008). Recent Pat. Eng..

[cit15] Bhattacharyya A., Kawi S., Ray M. (2004). Catal. Today.

[cit16] Gao B., Yap P., Lim T., Lim T. (2011). Chem. Eng. J..

[cit17] Xi Z., Ai Z., Jia F., Zhang L. (2008). J. Phys. Chem. C.

[cit18] Qin X., Cheng H., Wang W., Huang B., Zhang X., Dai Y. (2013). Mater. Lett..

[cit19] Xi Z., Ai Z., Jia F., Zhang L. (2008). J. Phys. Chem. C.

[cit20] Chen C. C., Fu J. Y., Chang J. L., Huang S. T., Yeh T. W., Hung J. T., Huang P. H., Liu F. Y., Chen L. W. (2018). J. Colloid Interface Sci..

[cit21] Liu H., Cao W., Su Y., Chen Z., Wang Y. (2013). J. Colloid Interface Sci..

[cit22] Novoselov K. S., Geim A. K., Morozov S. V., Jiang D., Zhang Y., Dubonos S. V., Grigorieva I. V., Firsov A. A. (2004). Science.

[cit23] Allen M., Tung V., Kaner R. (2010). Chem. Rev..

[cit24] Hou J., Yang C., Wang Z., Jiao S., Zhu H. (2013). Appl. Catal., B.

[cit25] Ai Z., Ho W., Lee S., Zhang L. (2009). Environ. Sci. Technol..

[cit26] Fu J. Y., Chen L. W., Dai Y. M., Liu F. Y., Huang S. T., Chen C. C. (2018). Mol. Catal..

[cit27] Ye L., Chen J., Tian L., Tiu J., Ppeng T., Deng K., Zan L. (2012). Appl. Catal., B.

[cit28] Ganesha R., Arivuoli D., Ramasamy P. (1993). J. Cryst. Growth.

[cit29] Yan H., Xu B., Shi S., Ouyang C. (2012). J. Appl. Phys..

[cit30] Solis-Pomar F., Martinez E., Melendrez M., Perez-Tijerina E. (2011). Nanoscale Res. Lett..

[cit31] Kang M., Seong K., Han J. K., Kim S. J., Chang S., Park C., Myung S. (2017). 2D Mater..

[cit32] Klemberg-Sapieha J., Oberste-Berghaus J., Martinu L., Blacker R., Stevenson I., Sadkhin G., Morton D., McEldowney S., Klinger R., Martin P., Court N., Dligatch S., Gross M., Netterfield R. (2004). Appl. Opt..

[cit33] Willey R. , Practical design and production of optical thin films, Taylor & Francis e-Library, Boca Raton, 2005

[cit34] Maurya M., Toutam V., Haranath D. (2017). ACS Omega.

[cit35] Yin P., Ling T., Lu Y., Xu Z., Qiao S., Du X. (2015). Adv. Mater..

[cit36] Lee W., Lu C., Chuang C., Chen Y., Fu J., Siao C., Chen C. (2015). RSC Adv..

[cit37] Thi Vu H., Atabaev T., Ahn J., Dinh N., Kim H., Hwang Y. (2015). J. Mater. Chem. A.

[cit38] Fakharuddin A., Ahmed I., Wali Q., Khalidin Z., Yusoff M., Rajan J. (2014). Adv. Mater. Res..

[cit39] Alekseev A., Lemmetyinen H., Tolkki A. (2015). J. Nanoelectron. Optoelectron..

[cit40] Mir W., Livache C., Goubet N., Martinez B., Jagtap A., Chu A., Coutard N., Cruguel H., Barisien T., Ithurria S., Nag A., Dubertret B., Ouerghi A., Silly M., Lhuillier E. (2018). Appl. Phys. Lett..

[cit41] Sfaelou S., Raptis D., Dracopoulos V., Lianos P. (2015). RSC Adv..

[cit42] Li X., Yu J., Wageh S., Al-Ghamdi A., Xie J. (2016). Small.

[cit43] Han M., Özyilmaz B., Zhang Y., Kim P. (2007). Phys. Rev. Lett..

[cit44] Chen Y., Crittenden J., Hackney S., Sutter L., Hand D. (2005). Environ. Sci. Technol..

[cit45] Ai Z., Ho W., Lee S. (2011). J. Phys. Chem. C.

[cit46] Zhang T. (2001). J. Photochem. Photobiol., A.

[cit47] Selvaraj R., Qi K., Jeong U., Al Nofli K., Al-Kindy S., Sillanpää M., Kim Y. (2015). Sultan Qaboos University Journal for Science [SQUJS].

[cit48] Lee W. W., Lu C. S., Chuang C. W., Chen Y. J., Fu J. Y., Siao C. W., Chen C. C. (2015). RSC Adv..

[cit49] Schneider C. A., Rasband W. S., Eliceiri K. W. (2012). Nat. Methods.

