# Supplementary material for: Photovoltaic and photocatalytic properties of bismuth oxyiodide–graphene nanocomposites
Source: RSC Adv. 2018 Dec 19;8(74):42254–61. doi: 10.1039/c8ra07360k (PMC9092079; doi:10.1039/c8ra07360k)
Supplement: RA-008-C8RA07360K-s001 [file RA-008-C8RA07360K-s001.pdf]

Electronic Supplementary Material (ESI) for RSC Advances.
This journal is © The Royal Society of Chemistry 2018

SUPPORTING INFORMATION

Photovoltaic and photocatalytic properties of bismuth oxyiodide-graphene nanocomposites

Levannie A. Mabuti,[a] Ian Kenneth S. Manding, [a] Candy C. Mercado [a*]

Department of Mining, Metallurgical, and Materials Engineering, University of the Philippines Diliman, 1101 Quezon City, Philippines

*Corresponding author e-mail: ccmercado1@up.edu.ph

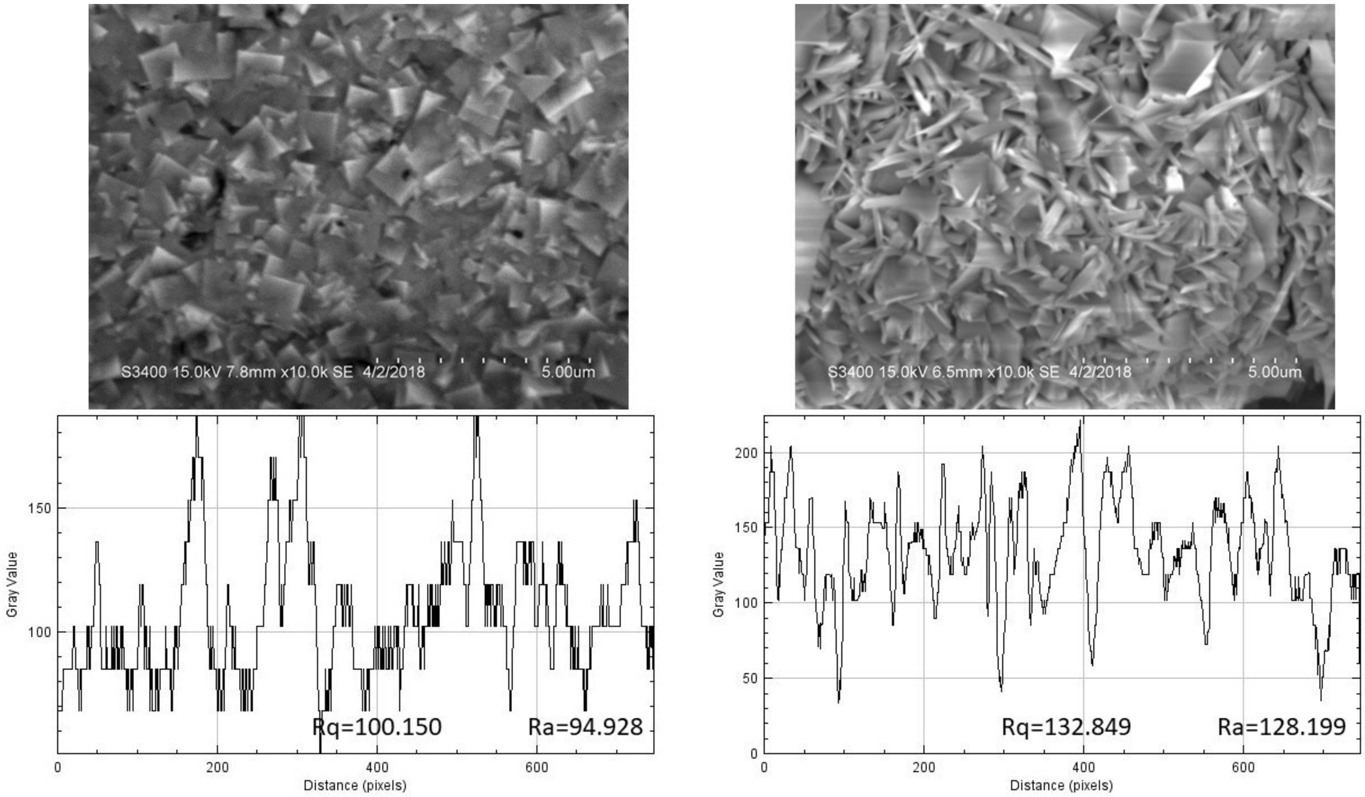

Figure S1. Roughness comparison from Image J roughness analysis show relative surface analysis of BiOI and BiOI-graphene SEM images. The roughness values taken was used to normalize the degradation percentage with respect to the total surface area available for the films.

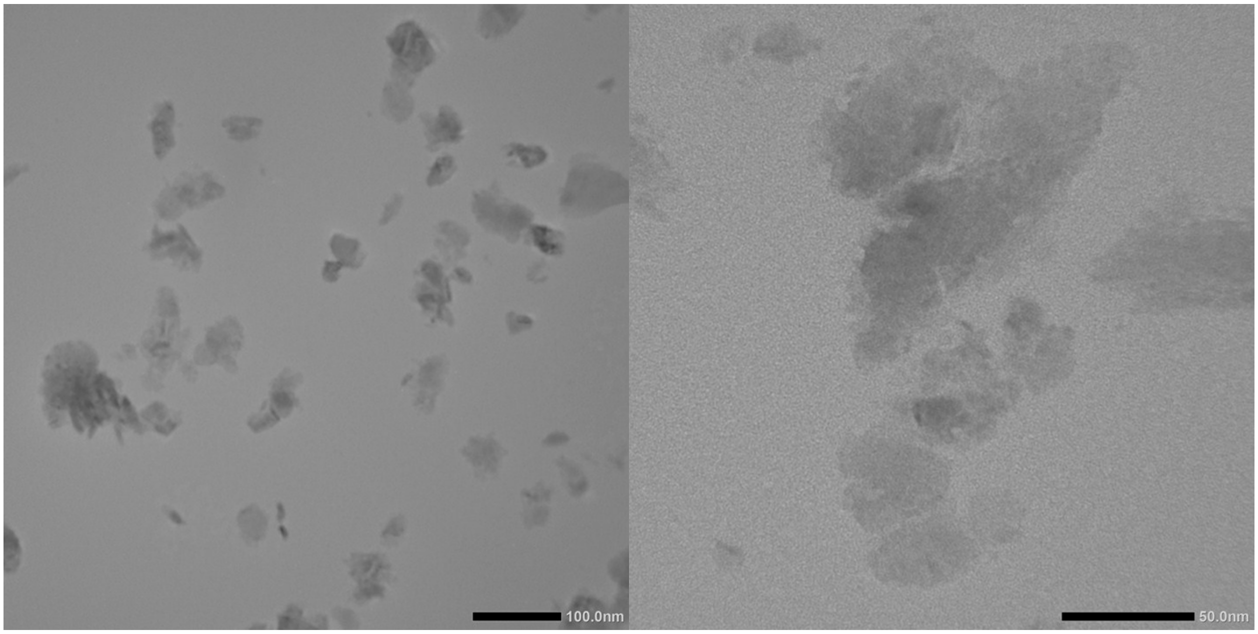

Figure S2. Transmission electron images of nano-BiOI after dispersion in water and ultrasonicated before mounting on TEM grids.

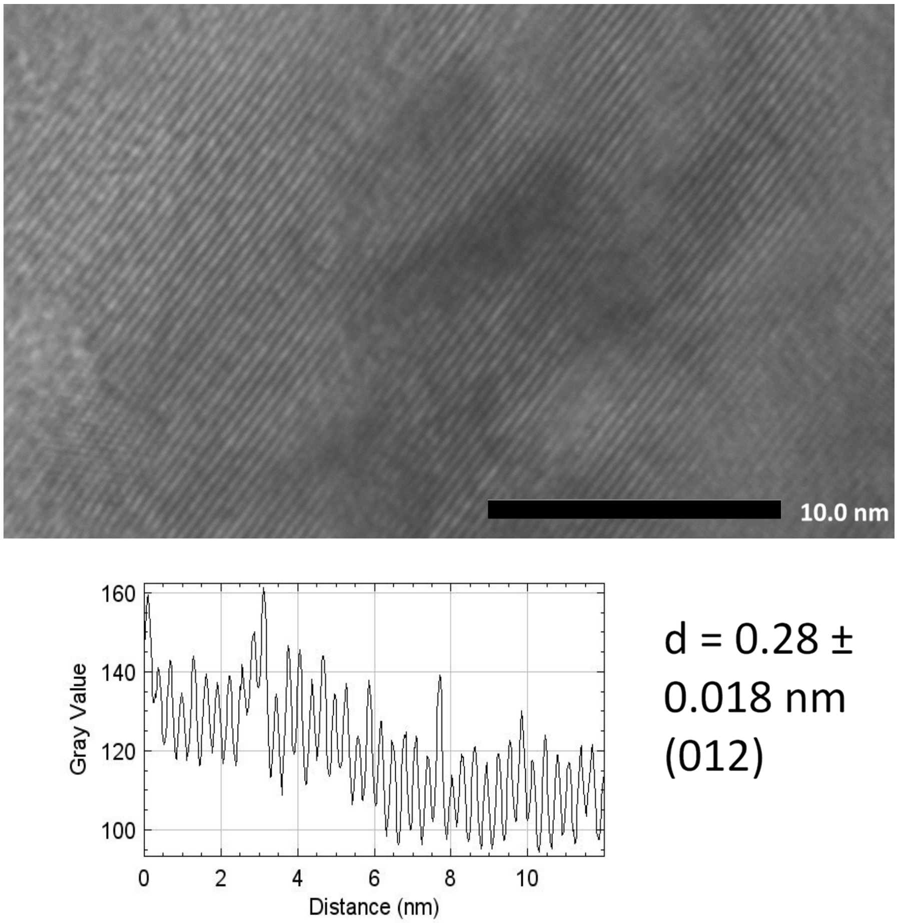

Figure S3. Analysis of the interatomic spacing using TEM and image processing through Image J. Distance (d) was the calculated peak to peak distance in the profile plot.

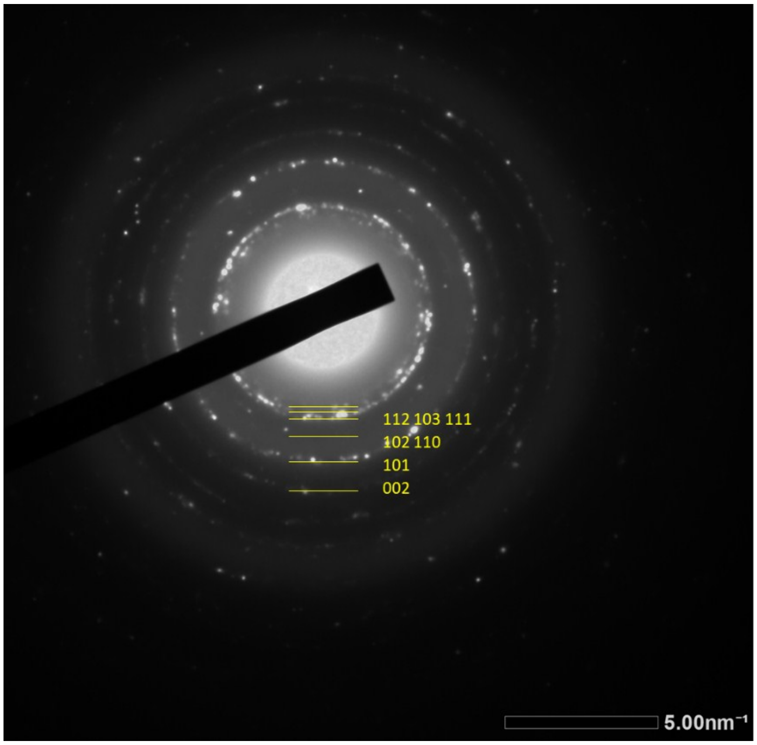

	2r	r (nm-1)	r/r0
1	6.22	3.11	1
2	6.39	3.195	1.027331
3	7.09	3.545	1.139871
4	8	4	1.286174
5	9.61	4.805	1.545016
6	11.77	5.885	1.892283
7	13.9	6.95	2.234727
8	15.3	7.65	2.459807
9	18.1	9.05	2.909968

hkl	r	r/r0
001	9.12	3.8159
002	4.56	1.90795
101	3.65	1.527197
102	3	1.25523
110	2.81	1.175732
111	2.69	1.125523
103	2.41	1.008368
112	2.39	1

Figure S4. Electron diffraction of BiOI nanoparticle wherein the polycrystalline diffraction patterns/spots were matched with the planes present in BiOI.

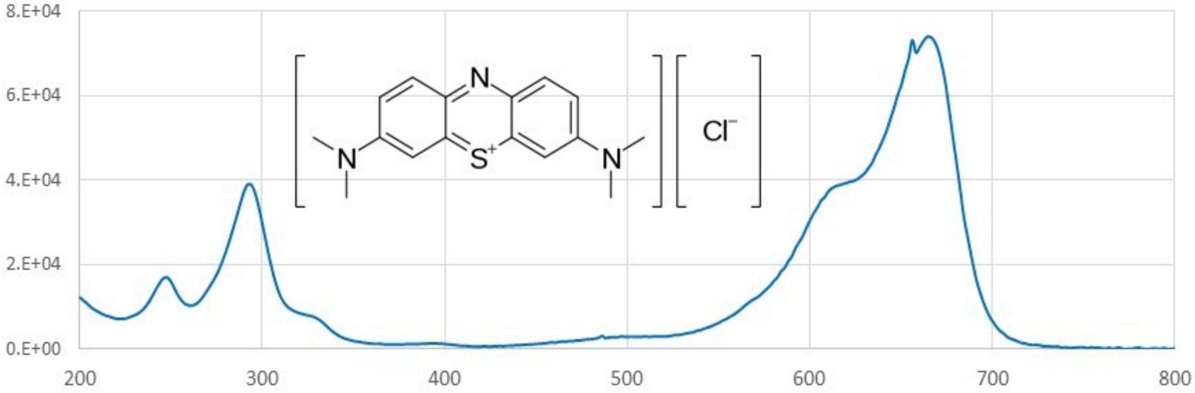

Figure S5. Molar extinction coefficient of methylene blue in water from [1].

a

d

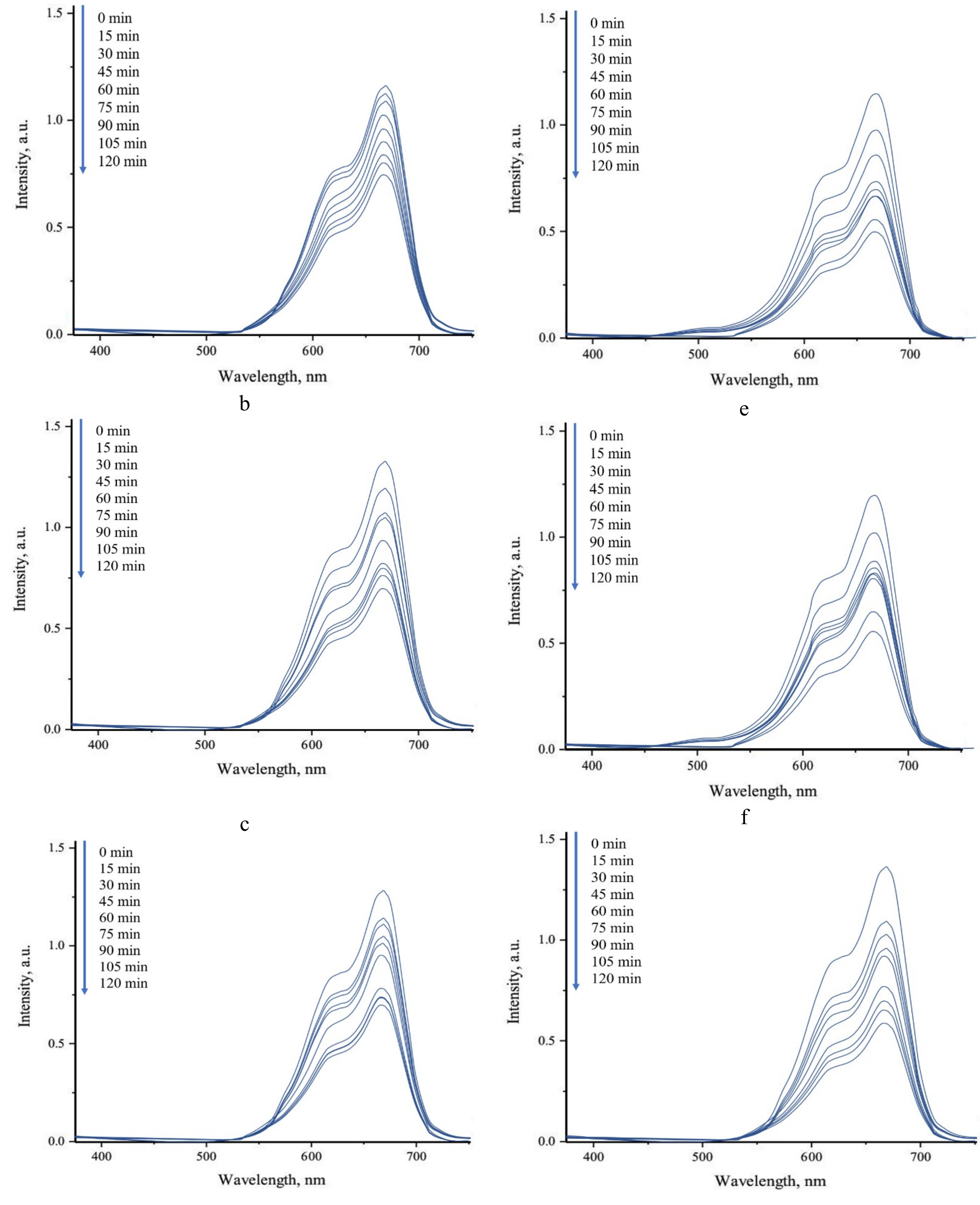

Figure S6. Degradation of methylene blue solution with immobilized BiOI film with deposition time a) 265, b) 280, c) 295 and BiOI-graphene composite film, deposition time d) 265, e) 280, f) 295 ^{o}C.

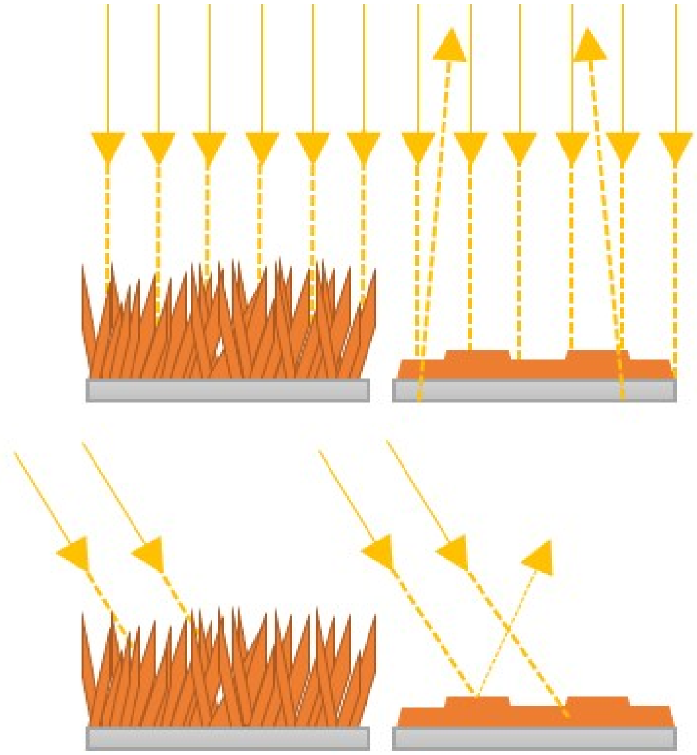

Figure S7. Nanoflake anti-reflectance (top) and light trapping (bottom).

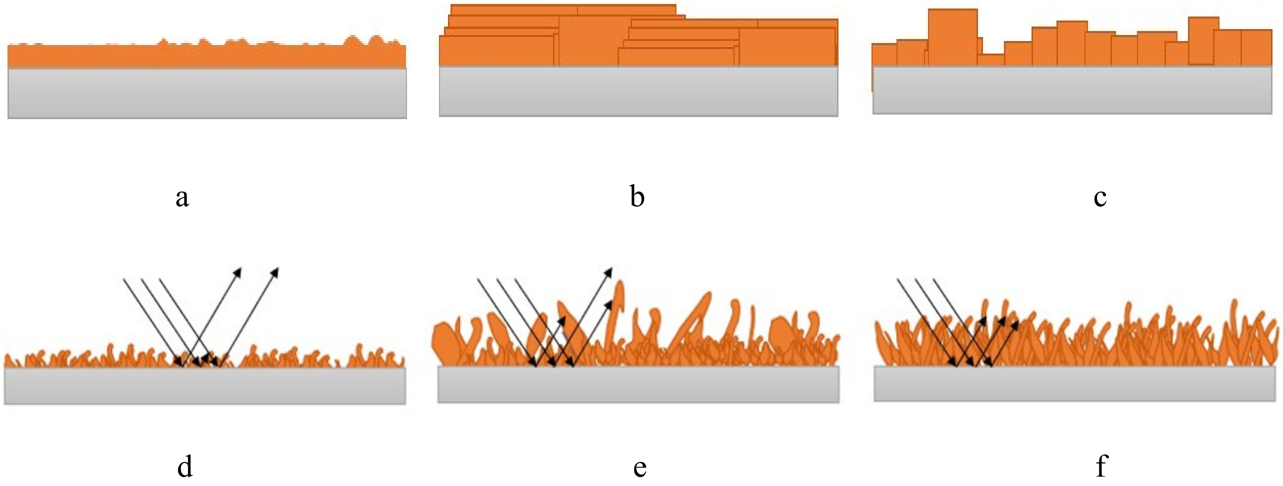

Figure S8. Visual representation of the light trapping of BiOI-265, BiOI-280, BiOI-295, BiOI-GR-265, BiOI-GR-280, BiOI- GR-295.

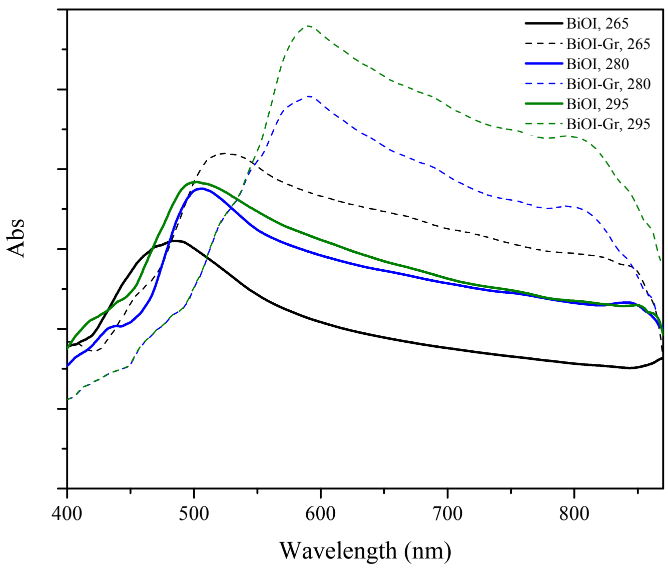

Figure S9. UV-vis absorbance of the films. The dip in the blue to UV region is due to the absorbance of the substrate (a fluorine doped tin oxide coated glass)

REFERENCES:

---

https://omlc.org/spectra/mb/mb-water.html
